# Socio-Economic Disparities in Use of Family Planning Methods among Pakistani Women: Findings from Pakistan Demographic and Health Surveys

**DOI:** 10.1371/journal.pone.0153313

**Published:** 2016-04-07

**Authors:** Syeda Kanwal Aslam, Sidra Zaheer, Muhammad Sameer Qureshi, Syeda Nisma Aslam, Kashif Shafique

**Affiliations:** 1 School of Public Health, Dow University of Health Sciences, Karachi, Pakistan; 2 Department of Biochemistry, Dow University of Health Sciences, Karachi, Pakistan; 3 Dr. Ishrat ul Ibad Institute of Oral Health Sciences, Dow University of Health Sciences, Karachi, Pakistan; 4 Institute of Health and Wellbeing, Public Health, University of Glasgow, 1-Lilybank Gardens, Glasgow, United Kingdom, G12 8RZ; Indiana University, UNITED STATES

## Abstract

**Background:**

Several developing countries like Pakistan step into Sustainable Development Goals period with crucial maternal and child health needs that need to be addressed for improving health outcomes among people. We aim to explore existent socio-economic disparities in use of family planning methods (FPM) among Pakistani women, and compare any such inequalities between the years 2006 and 2013.

**Setting:**

Pakistan Demographic and Health Surveys (PDHS) 2006–7 (n = 9177) and the most recent 2012–13(n = 13558) data were used to conduct secondary analysis. Participants were ever married women aged between 15 and 49 years. Socio-economic status was assessed by the education level and wealth index. Inequalities were measured through Odds Ratio (OR), Relative Index of inequality (RII), and Slope index of inequality (SII) on non-use of FPM.

**Results:**

Although the prevalence of FPM use has increased over time (28% in 2006 versus 54% in 2013), the socio-economic inequalities persistently exist. Comparing results of PDHS 2006 with PDHS 2013, education related absolute inequalities among urban dwellers increased from -0.41 (95% CI -0.67, -0.13, p-value < 0.01) to -0.83 (95% CI -1.02, -0.63, p-value < 0.01); and increased from -0.93 (95% CI -1.21, -0.64, p-value < 0.01) to -0.98 (95% CI -1.20, -0.76, p-value < 0.01) among rural dwellers. Similarly wealth related absolute inequalities are also existent.

**Conclusions:**

Although the FPM use has increased over time, but it is important to note that socio-economic gap in use of FPM persists. Such differences have disadvantaged the poor and the illiterate. Family planning programs may target the disadvantaged subgroups for ensuring well-being of women and children in Pakistan.

## Background

The world is poised to adapt the Sustainable Development Goals (SDG), and the SDG 3.7 calls for universal access to family planning services to ensure healthy lives and well-being [1]. However, several developing countries including Pakistan step into the post MDG period with national health profile that requires attention [2, 3]. The current estimates for Pakistan indicate that fertility rate decline has been slow, and current contraception use prevalence rate is 35%; unmet need of family planning is 20%, and fertility rate is 3.8 [4]. In the upcoming sustainable development discourse, utilization of family planning methods (FPM) thus remains crucial for improving well-being among Pakistanis [5]. The benefits include reduction of pregnancy related risks and adverse outcomes, improved sexual health, empowerment, and reducing early adolescent pregnancies among women; improved infant and child health outcomes, and prevention of unsustainable population growth [6]. Several socio-demographic factors including lack of education, low socio-economic status, and poor knowledge about family planning, are known determinants of family planning service utilization [7]. Various health system related factors like inadequate health services delivery, low access to outreach services, poor competence of family planning programs have also been linked with under-utilization of family planning services [7–9]. Therefore, it is important to realize that the socio-economic inequalities may exist in use of FPM, and addressing them remain important in order to achieve universal service utilization [10, 11]. Exploration of these factors in any given population is helpful in facilitating equity based efforts to improve use of FPM. Despite growing realization of the role of socio-economic characteristics in determining utilization of healthcare services, little is known about the existing socio-economic inequalities in use of family planning services. And majority of such contextual evidence has been reported from upper-middle and high income countries [12–14]. This would be the first study from Pakistan to report education and wealth based socio-economic inequalities using large data from nationally representative demographic health surveys. The objective of the study is to explore education and wealth related socio-economic gap in non-use of FPM by Pakistani population, and compare such inequalities between the year 2006 and year 2013.

## Methods

### Study design

Secondary analysis of nationally representative Pakistan Demographic and Health Survey (PDHS) 2006–07 and PDHS, 2012–13, was performed.

### Setting

The surveys were conducted in all urban and rural areas of Pakistan, a lower middle income, sixth most populous country in the world. The surveys aimed to collect information related to demographic, maternal and child health indicators.

### Participants

Ever married women aged between 15 and 49 years were interviewed. PDHS 2006–7: 10601 women were eligible, and 10023 completed the interview (response rate 94.5%) as indicated in PDHS report. The final n provided for data analysis is 9177. PDHS 2012–13: 14569 women were eligible. Among them 13558 completed the interview (response rate 93.1%), and is the final n used for data analysis.

### Data source

PDHS 2006–07 and PDHS, 2012–13 were conducted by the National Institute of Population Studies (NIPS), as part of the MEASURE DHS series, and the datasets of the two surveys are available upon request. (URL: http://dhsprogram.com/data/available-datasets.cfm). All details pertaining to the PDHS are available online, and only the important aspects are mentioned here [4, 15].

#### Sample size

The sampling frame consisted of Pakistani urban and rural areas. Two stage stratified sample design was used. PDHS 2012–13 used a nationally representative sample of 14,000 households from 500 primary sampling units (PSUs) was selected. PDHS 2006–7 sampled 10,601 women. For all details pertaining to sample design and implementation, please see PDHS 2012–13, and PDHS 2006–7 reports [4].

### Data collection tool

The pretested, structured, questionnaires used for PDHS were developed according to the standard MEASURE DHS program guidelines [16]. They were modified to suit Pakistani cultural context of family planning, maternal and child health, domestic violence, and HIV/AIDS related issues. The Married Woman’s Questionnaire collected the information from ever married women aged between 15–49 years.

### Ethical considerations

The standard MEASURE DHS guidelines regarding ethical considerations were followed to collect information for PDHS. Special care was taken while addressing sensitive issues like domestic violence among participant women [17, 18].

#### Statistical analyses

We used SAS version 9.1.3 for data analysis. Complex survey data analysis was used, as PDHS follows multistage cluster sampling design. Primary sampling units, final weights, and strata were used to adjust for cluster sampling. Chi-square test was used to determine significance of association between variables. Socio-economic inequalities were measured through Odds Ratios (OR), Relative Index of inequality (RII), andSlope index of inequality (SII) on non-use of FPM after adjusting for age. We performed separate analyses for urban and rural residence group; to compare the differences in non-use of FPM over the years 2006–7, and 2012–13.

### Independent variables

Socio-economic status was assessed by the wealth and education levels of the respondent.

Education level: PDHS obtained information related to the highest level of education obtained, and following categories were used:

No education: Participant confirmed that she never attended school.

Primary: Completed education up to class 5.

Secondary: Completed education up to class 10.

Higher: Refers to class 11 and above.

Wealth Index: PDHS used information related to household assets for creating wealth index. Firstly, wealth scores for all households were created by using a subset of indicators common to urban and rural households. Categorical variables were converted into separate dichotomous indicators. Principal component analysis was then used to produce a common factor score for each household. Secondly, area-specific indicators were used to create separate factor scores for urban and rural households. In third step, a nationally applicable wealth index was created by adjusting the separate area-specific factor scores through regression on the common factor score. The resultant combined wealth index has a mean of zero, and a standard deviation of one. Later, national level wealth quintiles are obtained by assigning household scores to each household member, to rank each person in the population by the score. Lastly the ranking is divided into five equal categories consisting of 20 percent of the population, namely: Poorest, Poorer, Middle, Richer, Richest [19].

### Dependent variable

Non-use of FPMwas measured by using the question: “Have you ever used any contraceptive method?” Response options were: Female sterilization, Male sterilization, Pill, IUD, Injectable, Condom, Lactational Amenorrhea (LAM), Emergency contraception, Standard days method (SDM), Rhythm, Withdrawal, Other, and No method. Option “no method” was considered as “non-use of FPM”. Report of any of the other options was recoded as “use of FPM”.

Regarding the measure of socio-economic dimensions of inequality, RII and SII have been considered as appropriate measures of health related inequalities in populations [20]. RII is computed by using estimates from the logistic regression, by ranking all participants according to their wealth index and education. A numerical measure of ranking between 0 and 1 is used for the level of education, and wealth index. Later, the ranked variables of education and wealth index are entered into logistic regression model as continuous covariates with “non-use of FPM” as the outcome, adjusting for age. RII can be interpreted as the prevalence ratio between the two ends of the education and wealth hierarchy [21]. The SII can be interpreted as absolute difference in the probability of reporting FPMnon-use (outcome variable) between the group with the lowest and the highest education and wealth ranks.

## Results

The PDHS 2012–13 was conducted among 13,558 Pakistani ever married women aged 15–49. Survey response rate was 93.1%. According to the results, overall prevalence ofFPMnon-use was 46% (n = 6204). The prevalence of FPM non-usewas 37.3% (n = 2372) among urban women and 53.2% (n = 3832) among rural women. Among urban population, 4.1% (n = 260) were in poorest, and 9.1% (n = 576) were poorer; and 39.4% (n = 2503) had no education, and 14.1% (n = 893) had primary education. Among rural population, 30.9% (n = 2226) were in poorest, and 27.9% (n = 2010) were poorer; and 71.1% (n = 5122) had no education, and 13% (n = 938) had primary education. Comparing the descriptiveresults with those of the 2006–7 survey, the prevalence of FPM non-use was 72.3% overall, 78.6% among rural, and 60.3% among urban dwellers.

Comparing the FPM non-use among different education quintiles, 44.9% (n = 1125) of illiterate urban women, and 57.3% (n = 2933) of illiterate rural women reported FPM non-use in 2013. As far as wealth related disparities are concerned, 50.4% (n = 131) of urban poorest women, and 68.5% (n = 1525) of rural poorest women reported FPM non-use in 2013. (Table 1)

**Table 1:** Descriptives of FPM use according to socio-demographic characteristics among ever-married Pakistani women.

	PDHS 2006–7 (n = 9177)						PDHS 2012–13 (n = 13588)					
	Urban			Rural			Urban			Rural		
	FPM use						FPM use					
	Yes	No	p-value*	Yes	No	p-value*	Yes	No	p-value*	Yes	No	p-value*
**Educational level n (%)**												
No education	490 (32.0)	1041 (68.0)	< 0.01	876 (18.8)	3784 (81.2)	< 0.01	1378 (55.1)	1125 (44.9)	< 0.01	2189 (42.7)	2933 (57.3)	< 0.01
Primary	201 (42.0)	278 (58.0)		222 (28.6)	555 (71.4)		579 (64.8)	314 (35.2)		546 (58.2)	392 (41.8)	
Secondary	325 (45.0)	398 (55.0)		141 (30.7)	318 (69.3)		1060 (66.9)	524 (33.1)		457 (55.0)	374 (45.0)	
Higher	232 (56.3)	180 (43.7)		51 (37.5)	85 (62.5)		962 (70.2)	409 (29.8)		183 (57.9)	133 (42.1)	
**Wealth Index n (%)**												
Poorest	26 (19.1)	110 (80.9)	< 0.01	243 (12.2)	1757 (87.8)	< 0.01	129 (49.6)	131 (50.4)	< 0.01	701 (31.5)	1525 (68.5)	< 0.01
Poorer	68 (21.7)	246 (78.3)		302 (18.1)	1369 (81.9)		291 (50.5)	285 (49.5)		951 (47.3)	1059 (52.7)	
Middle	176 (29.0)	430 (71.0)		348 (27.9)	898 (72.1)		581 (54.8)	480 (45.2)		831 (54.4)	697 (45.6)	
Richer	366 (41.4)	517 (58.6)		255 (32.8)	523 (67.2)		1064 (63.6)	609 (36.4)		588 (59.8)	396 (40.2)	
Richest	612 (50.7)	594 (49.3)		142 (42.1)	195 (57.9)		1914 (68.8)	867 (31.2)		304 (66.2)	155 (33.8)	

FPM: Family planning methods; PDHS: Pakistan Demographic and Health Survey

*p-value: calculated by using Chi-square test for categorical variables

Further, results of age-adjusted Odds ratio also indicate thatodds of FPM non-use increases with decreasing education[Urban 2013, No education: OR = 2.15 (95% CI 1.86, 2.48), Rural 2013, No education: OR = 2.45 (95% CI -1.93, 3.10)]. Similarly odds of FPM non-use increase with decreasing wealth index[Urban 2013, Poorest: OR = 2.13 (95% CI 1.64, 2.76), Rural 2013, Poorest: OR = 4.14 (95% CI 3.33, 5.14)]. (Table 2)

**Table 2:** Odds ratio and 95% confidence interval of FPM non-use according to socio-demographic characteristics among ever-married women.

Variables	FPM non-use							
	PDHS 2006–7 (n = 9177)				PDHS 2012–13 (n = 13588)			
	Urban		Rural		Urban		Rural	
	OR (95% CI)	aOR (95% CI)	OR (95% CI)	aOR (95% CI)	OR (95% CI)	aOR (95% CI)	OR (95% CI)	aOR (95% CI)
**Educational level**								
No education	2.73 (2.19–3.42)	2.85 (2.28–3.56)	2.59 (1.81–3.69)	2.81 (1.96–4.02)	1.92 (1.66–2.20)	2.15 (1.86–2.48)	1.84 (1.46–2.32)	2.45 (1.93–3.10)
Primary	1.78 (1.36–2.32)	1.72 (1.32–2.25)	1.50 (1.02–2.19)	1.39 (0.94–2.04)	1.27 (1.06–1.52)	1.21 (1.01–1.46)	0.98 (0.76–1.27)	1.06 (0.81–1.38)
Secondary	1.57 (1.23–2.01)	1.55 (1.20–1.96)	1.35 (0.90–2.01)	1.19 (0.79–1.78)	1.16 (0.99–1.35)	1.06 (0.90–1.24)	1.12 (0.86–1.45)	1.08 (0.83–1.41)
Higher	Ref	Ref	Ref	Ref	Ref	Ref	Ref	Ref
**Wealth Index**								
Poorest	4.35 (2.80–6.78)	4.54 (2.91–7.08)	5.26 (4.08–6.79)	5.92 (4.57–7.67)	2.24 (1.73–2.89)	2.13 (1.64–2.76)	4.26 (3.44–5.27)	4.14 (3.33–5.14)
Poorer	3.72 (2.78–4.98)	3.79 (2.83–5.08)	3.30 (2.57–4.23)	3.52 (2.73–4.54)	2.16 (1.80–2.59)	2.06 (1.71–2.48)	2.18 (1.76–2.70)	2.11 (1.70–2.62)
Middle	2.51 (2.04–3.10)	2.57 (2.09–3.18)	1.87 (1.46–2.41)	1.98 (1.54–2.55)	1.82 (1.57–2.10)	1.71 (1.47–1.97)	1.64 (1.32–2.04)	1.58 (1.26–1.97)
Richer	1.45 (1.22–1.73)	1.47 (1.23–1.75)	1.49 (1.14–1.94)	1.55 (1.19–2.03)	1.26 (1.11–1.43)	1.19 (1.04–1.36)	1.32 (1.04–1.66)	1.24 (0.98–1.57)
Richest	Ref	Ref	Ref	Ref	Ref	Ref	Ref	Ref

OR: crude odds ratio, aOR: adjusted for age, FPM: Family planning methods, PDHS: Pakistan Demographic and Health Survey.

Results of 2012–13 survey indicate existence of socio-economic inequality in FPM non-use among Pakistani population, disadvantaging the less educated and the poor. Regarding the results of urban population, the less educated [Urban 2013: RII = 2.75 (95% CI 2.55, 2.94), SII = -0.83 (95% CI -1.02, -0.63)]; and the poor women [Urban 2013: RII = 2.60 (95% CI 2.40, 2.79), SII = -0.77 (95% CI-0.96, -0.57)] were disadvantaged. Similarly, among rural population, the less educated [Rural 2013: RII = 2.27 (95% CI 2.04, 2.48), SII = -0.98 (95% CI -1.20, -0.76)], and the poor women [Rural 2013: RII = 2.71 (95% CI 2.53, 2.88), SII = -1.33 (95% CI -1.50, -1.15)] were disadvantaged. Education related gap is wider among urban population as compared to rural population [Urban RII = 2.75, as compared to Rural RII = 2.27). (Table 3)

**Table 3:** Logistic regression-based age-adjusted Relative and Absolute Indices of Inequality (RII and SII) for FPM non-use.

Variables	PDHS 2006–7 (n = 9177)		PDHS 2012–13 (n = 13588)	
	Urban	Rural	Urban	Rural
**Education**				
RII	1.90 (1.63,2.16)	1.71 (1.43,1.99)	2.75 (2.55,2.94)	2.27 (2.04,2.48)
SII	- 0.41 (-0.67,-0.13)	-0.93 (-1.21,-0.64)	-0.83 (-1.02,-0.63)	-0.98 (-1.20,-0.76)
p-value	< 0.01	< 0.01	< 0.01	< 0.01
**Wealth Index**				
RII	2.93 (2.64,3.21)	1.99 (1.76,2.23)	2.60 (2.40,2.79)	2.71 (2.53,2.88)
SII	-1.45 (-1.73,-1.16)	-1.62 (-1.85,-1.38)	-0.77 (-0.96,-0.57)	-1.33 (-1.50,-1.15)
p-value	< 0.01	< 0.01	< 0.01	< 0.01

RII: relative index of inequality (95% confidence intervals). SII: Slope index of inequality (95% confidence intervals). FPM: Family planning methods; PDHS: Pakistan Demographic and Health Survey

Comparing the results of the present survey with PDHS 2006–7, the education related socio-economic inequalities have generally increased further in 2013 as compared to 2006. Currently, the education related gap is widest among urban population, and has increased from 1.90 in 2006 [RII = 1.90 (95% CI 1.63, 2.16), SII = -0.41 (95% CI -0.67, -0.13)] to 2.75 in 2013. Wealth related inequalities among urban population are still existent in 2013 [RII = 2.60 (95% CI 2.40, 2.79), SII = -0.77 (95% CI -0.96, -0.57)], and have decreased from 2006 [RII = 2.93 (95% CI 2.64, 3.21), SII = -1.45 (95% CI -1.73, -1.16)]. (Table 3)

## Discussion

Our study indicates existence of socio-economic inequality in FPM use in Pakistan in most recent period (2012–2013); and the poorer and less educated women are disadvantaged in this regard. Comparing the results of the present survey with those of 2006–07, it is very important to note that the education related inequality gap has widened further over the study period; and currently the gap is widest among urban population across different education categories. Wealth related inequalities among urban population are also still existent in the year 2013.

Almost half of all married women in reproductive age have never used any contraceptive methods and the prevalence estimates remain higher than many developing countries with similar or worse socio-demographic profiles [22]. Regionally, the proportion of women aged 15–49 reporting use of a modern contraceptive method in Asia is 62% (prevalence of non-use is 38%)[23, 24]. The use of the services has increased over the concerned study period (2006 to 2013), which might be expected owing to various governmental and non-governmental interventions that have been introduced over time. However the main focus of the study was to examine any existent disparities in use with respect to wealth and education. And we observed that improvement has occurred over time, but is not consistent across different categories of defined characteristics including wealth, education, and urban/rural way of living, and thus highlights the existence of socioeconomic disparities. Results of the PDHS 2012–13 also indicate that twenty percent of the married women’s family planning services need has been unmet, and the very fact highlights the underlying multifaceted social, and system related factors that may have resulted in underperformance of the country’s family planning programs [4, 25]. As far as the education related disparities in FPM non-use is concerned, it is alarming that such disparities are widening further; and the gap has nearly doubled between the years 2006 and 2013. More so, the findings of this study indicate that women with no education remain the major contributors of the FPM non-use. At this point in time, however, when the country’s reproductive health indicators remain low, (contraception use prevalence rate is 35%, unmet need of family planning is 20%, and fertility rate is 3.8) it may be imperative to prioritize the issue of existing socio-economic disparities [4]. It thus holds importance in planning the discourse of upcoming SDG implementation in country’s policies as well. This implies that if the SDG 3.7 (aiming at achieving universal access to reproductive health services) has to be implemented with the aim of achieving real progress in improving well-being of Pakistani mothers and children; then the target of reducing education related disparities becomes more necessarynow than ever to specifically design equity based family planning programs addressing the needs of illiterate women.

Further, we noted that such education related disparity gap is wider among urban females compared with those residing in rural settings of Pakistan. It is generally perceived that rural populations are at a disadvantage when it comes to health services, but findings of this study highlight that as far as FPM non-use is concerned, the disparities are more pronounced among urban populations. On the contrary, wealth related gap has closed further over the 2006–13 period, but is still existent. There may be several complex phenomena that may have played their role in this regard. On one side, World Health Organization reports that globally, unmet need for family planning is particularly high among subpopulations like, migrants, urban slum dwellers, and refugees [26]. The very same finding may explain the existent socio-economic gap in non-use of FPM among Pakistani urban population. It is important to note here, that Pakistan (previously an agrarian society) faces the issue of urban-rural migration (internal migration); as people shift to industrialized and business oriented urban cities for better economic prospects. And many of the big cities are undergoing rapid urbanization, with ever swelling urban populations [27–29]. Further, there are other specific socio-political aspects to be considered here: Pakistan currently stands as the front face on war on terrorism, and several of the internally displaced families from areas subjected to extreme levels of civil unrest, and from war affected regions like Afghanistan take refuge in the urban cities due to economic feasibilities [29–31]. They seek better economic prospects through migration to bigger urbanized cities. These subgroups of populations generally exhibit low socio-economic profile.Such conflict affected families may be more prone to health related disparities [32, 33]. However, on the other side, various governmental and non-governmental programs have introduced maternal and child health interventions targeting reproductive health of Pakistani women. These efforts have been targeted towards the vulnerable subgroups. This may offer some explanation for the closing of wealth related disparities in FPM use among urban dwellers over the 2006–13 period. Nevertheless, the health system may still be failing to completely reach and care for the vulnerable subgroups. Could this explain the existing health related disparities among Pakistani women? We need more evidence to validate these speculations, and explore the issue further.

Our study had various limitations. Firstly, cross-sectional nature of the study design, which hampers the establishment of temporal association. It might be possible that FPM non-use has led to lower socio-economic status among the respondents. Although education is taken as an indicator of socio-economic position in this study, and for majority of participants, it may hold true that formal education received in earlier years of life was reported, nevertheless, the limitation of establishing temporal association has to be acknowledged. Secondly, the study concentrates on particular aspects of education and wealth as the causative variables, as has been the major focus in several previous research studies focusing on socioeconomic disparities. Nevertheless, various other important factors including regions/provinces and ethnicity may also have some effects on the outcome. We could not address the issue of non-responders as this was a secondary analysis; and the results may have be biased. However, given large sample size of the study, it may hold true that findings are representative for majority of the population. The study contributes to the limited evidence available on socio-economic inequalities related to FPM use in developing countries. To the best of our knowledge this is the first contextual study from the country to report education and wealth related inequalities using relative and slope inequality index. We used recent PDHS data which provides nationally representative results, using sound methodological approaches.

## Conclusion

Although the use of FPM has increased over time, but the socio-economic gap in FPM non-use persistently exists. Such differences have disadvantaged the poor and the illiterate, and are more eminent among the different education quintiles of urban residents. Family planning programs may use this information to devise improved programs targeting the disadvantaged subgroups for improved use of family planning services.
